# Supplementary material for: TERT Promoter and BRAF V600E Mutations in Papillary Thyroid Cancer: A Single-Institution Experience in Korea
Source: Cancers (Basel). 2022 Oct 8;14(19):4928. doi: 10.3390/cancers14194928 (PMC9563418; doi:10.3390/cancers14194928)
Supplement: Supplementary file 1 [file cancers-14-04928-s001.zip › cancers-1948145-supplementary.pdf]

Table S1. Baseline characteristics.

Characteristics	No. (n = 7797)
Age (yr)	43.25 ± 12.20
<55	6250 (80.2)
≥55	1547 (19.8)
Sex	
Male	1804 (23.1)
Female	5993 (76.9)
Tumor size (cm)	0.91 ± 0.69
≤1.0	5592 (71.7)
>1.0	2205 (28.3)
Multiplicity	
Single	5215 (66.9)
Multiple	2582 (33.1)
Extrathyroidal extension	
Absent	7062 (90.6)
Present	735 (9.4)
Histology	
Classic	7182 (92.1)
Follicular variant	405 (5.2)
Tall-cell variant	79 (1.0)
Others	131 (1.7)
Histology	
Classic	7182 (92.1)
Variant	615 (7.9)
BRAF V600E	
Absent	1251 (16.0)
Present	6546 (84.0)
Perinodal infiltration	
Absent	7066 (90.6)
Present	731 (9.4)
T stage	
T1	6794 (87.1)
T2	242 (3.1)
T3	528 (6.8)
T4	233 (3.0)
T stage	
T1-T2	7036 (90.2)
T3-T4	761 (9.8)
N stage	
N0	4311 (55.3)
N1a	2716 (34.8)
N1b	770 (9.9)
N stage	
N0	4311 (55.3)
N1a-N1b	3486 (44.7)
M stage	
M0	7774 (99.7)
M1	23 (0.3)

TNM stage	
I	6561 (84.1)
II	1164 (14.9)
III	59 (0.8)
IV	13 (0.2)
TNM stage	
I-II	7725 (99.1)
III-IV	72 (0.9)
Radioactive iodine therapy	
No	6311 (80.9)
Yes	1486 (19.1)

Values are expressed as the mean ± standard deviation or as numbers (%).
